# Triethylene glycol, an active component of Ashwagandha (*Withania somnifera*) leaves, is responsible for sleep induction

**DOI:** 10.1371/journal.pone.0172508

**Published:** 2017-02-16

**Authors:** Mahesh K. Kaushik, Sunil C. Kaul, Renu Wadhwa, Masashi Yanagisawa, Yoshihiro Urade

**Affiliations:** 1 Department of Molecular Sleep, International Institute for Integrative Sleep Medicine (WPI-IIIS), University of Tsukuba, 1-1-1 Tennodai, Tsukuba, Ibaraki, Japan; 2 Department of Molecular Genetics, International Institute for Integrative Sleep Medicine (WPI-IIIS), University of Tsukuba, 1-1-1 Tennodai, Tsukuba, Ibaraki, Japan; 3 Drug Discovery and Assets Innovation Lab, DBT-AIST International Laboratory for Advanced Biomedicine (DAILAB), National Institute of Advanced Industrial Science and Technology, Higashi 1-1-1, Tsukuba, Ibaraki, Japan; Torrey Pines Institute for Molecular Studies, UNITED STATES

## Abstract

Insomnia is the most common sleep complaint which occurs due to difficulty in falling asleep or maintaining it. Most of currently available drugs for insomnia develop dependency and/or adverse effects. Hence natural therapies could be an alternative choice of treatment for insomnia. The root or whole plant extract of Ashwagandha (*Withania somnifera*) has been used to induce sleep in Indian system of traditional home medicine, Ayurveda. However, its active somnogenic components remain unidentified. We investigated the effect of various components of Ashwagandha leaf on sleep regulation by oral administration in mice. We found that the alcoholic extract that contained high amount of active withanolides was ineffective to induce sleep in mice. However, the water extract which contain triethylene glycol as a major component induced significant amount of non-rapid eye movement sleep with slight change in rapid eye movement sleep. Commercially available triethylene glycol also increased non-rapid eye movement sleep in mice in a dose-dependent (10–30 mg/mouse) manner. These results clearly demonstrated that triethylene glycol is an active sleep-inducing component of Ashwagandha leaves and could potentially be useful for insomnia therapy.

## Introduction

Insomnia and other sleep disorder such as restless leg syndrome are common complaints among the middle-aged population. Insomnia is one of the most common neuropsychiatric disorders, with an estimated incident of 10–15% in general population and 30–60% in elderly population [1]. It is closely linked with certain other diseases including obesity, cardiovascular diseases, depression, anxiety, mania deficits etc., due to behavioral, hormonal and neurochemical alternations [2, 3]. It also causes huge economic loss to the society by means of traffic accident, less productivity at work and clinical cost for the treatment. Most commercially available drugs that are used to treat insomnia are based on benzodiazepines, modulation of melatonin or histamine based mechanism. These drugs are often associated with major or minor adverse effects that includes dependency, withdrawal symptoms or other side effects [4–8]. Recently approved orexin receptor-based insomnia therapy, suvorexant (Balsomra®), also showed side effects [9]. Hence, natural compounds with sleep-inducing potential are an alternative to avoid side effects and dependency of those synthetic drugs that are currently used to treat insomnia.

Ashwagandha (*Withania somnifera*) is a central herb in Ayurveda, the traditional medicine system native to India. It has been used for thousands of years in Ayurvedic medicine and classified as a *Rasayana* (chemical) herb that promotes body- and brain-health by multiple ways including, increasing the stress tolerance and strengthening the immune system [10]. As signified by its name “somnifera- meaning sleep-inducing”, it has been recommended for sound sleep through centuries. It is believed that Ashwagandha is an adaptogen (natural compound that help the body adapt to stress) that corrects the imbalance between immune and neuroendocrine system to normalize the bodily functions under stressed conditions by targeting hypothalamic-pituitary-adrenal gland axis [11]. Pre-treatment with Ashwagandha extract has protective effects against stress, cancer and age-associated neurodegenerative conditions [12–15]. The root extract administration induced sleep in rats after oral administration [16, 17]. However, those previous studies used root extracts that can have slightly overlapping but different composition with that of our study. Moreover, the active component of Ashwagandha, which is responsible for sleep induction was still remain unidentified. Based on current available studies, we analyzed the effect of alcoholic and water extracts of Ashwagandha leaves on sleep quality and quantity by recording electroencephalogram (EEG) and electromyogram (EMG) in mice. We found that the triethylene glycol (TEG) in the water extract induced sleep in mice in a dose-dependent manner and is a natural sleep-inducing molecule.

## Materials and methods

### Animals

We used male C57BL/6 mice weighing 24–30 g (11–13 weeks) for experiments. C57BL/6 mice were obtained from Japan SLC (Shizuoka, Japan), and housed in an insulated sound-proof recording room maintained at an ambient temperature of 23 ± 0.5°C with a relative humidity of 50 ± 5% on an automatically controlled 12 h light/dark cycle (light on at 0500; illumination intensity above 100 lux). Mice were fed *ad libitum* and given sufficient water. The experimental protocols were approved by the University of Tsukuba Animal Ethics Committee, and every effort was made to minimize the number of animals used as well as any pain and discomfort (Animal ethical approval number; 16086).

### Surgery

Under anesthesia using pentobarbital (50 mg/kg, intraperitoneally) mice were chronically implanted with EEG and EMG electrodes for polysomnography, as previously described [18]. Briefly, the implant consisted of 2 stainless steel screws (1 mm diameter) serving as EEG electrodes, one of which was placed epidurally over the right frontal cortex (1.0 mm anterior and 1.5 mm lateral to bregma) and the other over the right parietal cortex (1.0 mm anterior and 1.5 mm lateral to lambda). Two Teflon coated stainless steel wires (0.2 mm in diameter), which were placed bilaterally into the trapezius muscles, served as EMG electrodes. Both EEG and EMG electrodes were connected to a micro connector. The whole assembly was then fixed to the skull with self-curing dental acrylic resin. Antibiotic and analgesic drugs were administered up to 5 days post-operatively.

### EEG/EMG recording and analysis

After 8–10 days of post-operative recovery, the mice were placed in experimental cages for a 4-day habituation/acclimatization period and connected with counterbalanced recording leads. Baseline EEG/EMG was recorded for 24 h starting onset of dark phase. All mice that were subjected to EEG/EMG recordings received vehicle and multiple doses of Ashwagandha leaf extracts or pure TEG (10 mg, 20 mg, and 30 mg/mouse) on different days and any two administrations were separated by, at least, 2 days. Each EEG/EMG recording and drug administration was performed *per os* (*p*.*o*.), at the onset of dark phase (17:00 h). Cortical EEG and EMG signals were amplified and filtered (EEG, 0.5–30 Hz; EMG, 20–200 Hz), then digitized at a sampling rate of 128 Hz, and recorded by using SleepSign software (Kissei Comtec, Nagano, Japan) as previously described [19]. Polysomnographic recordings were scored with automated analysis, off-line, in 10-s epochs as wakefulness, rapid eye movement (REM) and non-REM (NREM) sleep by SleepSign software, using standard criteria [19, 20]. The defined sleep–wake stages were visually examined and corrected, wherever necessary. Spectral analysis of EEG by fast Fourier transformation (FFT) was performed, and the EEG power densities of each 0.5- Hz bin were averaged by calculating the percentage of each bin with respect to the total power in the range of 0.5–35 Hz.

### Preparation of leaf extract of Ashwagandha

Ashwagandha plants were raised in Nihari-mura, Ibaraki, Japan and kindly provided for experimental use. Alcoholic extract of Ashwagandha leaves were prepared as described previously [21, 22]. Briefly, the leaves of Ashwagandha were washed, air-dried and ground to a fine powder. The fine leaf powder was extracted by Soxhlet method. The leaf powder (40 g) was exhaustively extracted with methanol (60°C) in Soxhlet apparatus for 100–110h. Methanol was distilled-off to concentrate the extract. It was made aqueous by adding double-distilled water and was then extracted with hexane to remove chlorophyll and other pigments. Residual aqueous methanolic solution was re-extracted with diethyl ether using separating funnel. Diethyl ether was evaporated and the extract was solubilized in sterile DMSO for use. Water extract was prepared as described previously [15]. For cyclodextrin-assisted aqueous extraction of Ashwagandha leaves, the dried leaf powder (10% w/v) was mixed with aqueous solution of 7.5% gamma-cyclodextrin. The mixture was stirred for 24 h at 37°C with slow shaking (90 rpm) in TAITEC Bio-Shaker BR-43FL. The slurry was centrifuged at 3500 rpm for 10 min and the supernatant was filtered through 0.45-micron filter.

### Statistical analysis

All data were expressed as mean ± SEM. The statistical significance of time-course changes for sleep-wake profiles and power density were analyzed by paired t-test. Dose dependency data of wake, REM and NREM sleep, number of sleep-wake bouts, episode duration and stage transition were analyzed using one-way ANOVA followed by Least Square Difference (LSD) post-hoc test. In all of the cases, p<0.05 was considered as significant.

## Results

### TEG, but not withanone or withaferin A, caused induction of sleep in mice

**Fig 1:**
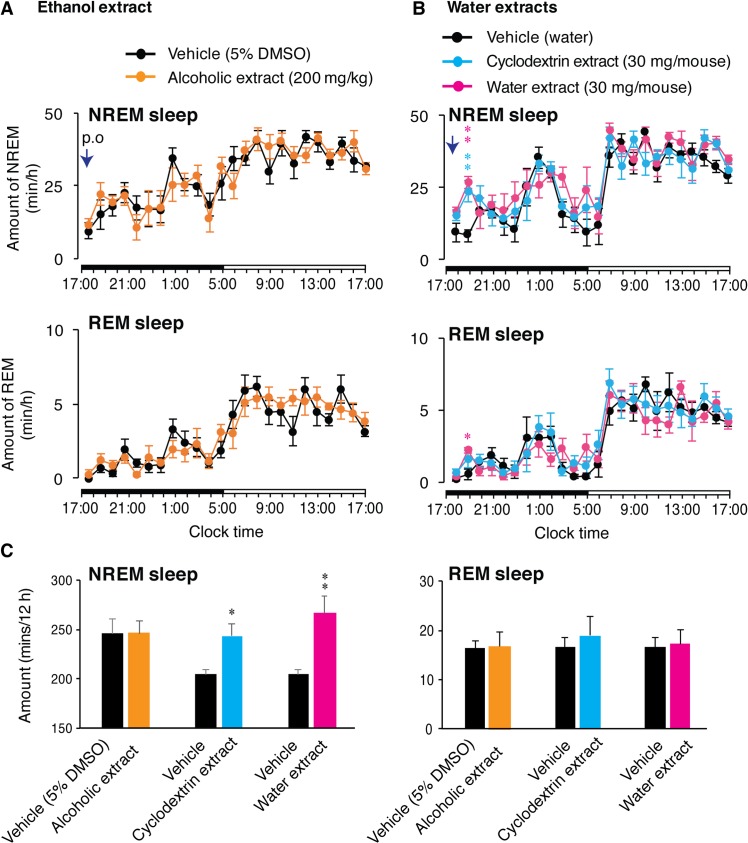
Oral administration of various extracts from Ashwagandha leaves in mice and their effect on sleep-wakefulness. (A) Hourly plots of NREM and REM sleep after *p*.*o*. administration of vehicle (black circles) or alcoholic extract of Ashwagandha leaves (orange circles; n = 5). (B) Hourly plots of NREM and REM sleep after *p*.*o*. administration of vehicle (black circles) or water extracts of Ashwagandha leaves; cyclodextrin extract (blue circle) and water extract (magenta circles; n = 6). Arrows indicate time of *p*.*o*. administration. Black and white horizontal bars indicate 12 h dark and 12 h light period. (C) Changes in total amount of NREM (left graph) and REM sleep (right graph) during dark phase after *p*.*o*. administration of vehicle (black bars) and various Ashwagandha leaf extracts (colored bars). All administrations were done at the onset of dark period (17:00 h). Data presented as mean ± SEM; *p≤0.05, **p≤0.01 vs vehicle by using paired t-test.

Ashwagandha leaf extracts were prepared via two methods viz alcohol-based (Ethanol) extraction and water based-extraction (named; water & cyclodextrin extracts). These extracts were tested for their sleep-inducing properties in mice after *p*.*o*. administration followed by EEG/EMG recordings. Oral administration of the ethanol extract failed to change either NREM or REM sleep in mice (Fig 1A). Total amount of REM and NREM sleep remain indistinguishable between vehicle (5% DMSO) and ethanol extract (200 mg/kg) administration (Fig 1C), suggesting that withanolides (withaferin A and withanone), major components of the leaf extract, can be ruled out for the sleep-inducing property of Ashwagandha. On the other hand, both water and cyclodextrin extracts, increased NREM sleep significantly as compared to vehicle administration (Fig 1B). Total amount of NREM sleep over 12 h was also significantly increased after administration of those extracts (Fig 1C).

### TEG-induced NREM, but not REM, sleep in wild-type mice

**Fig 2:**
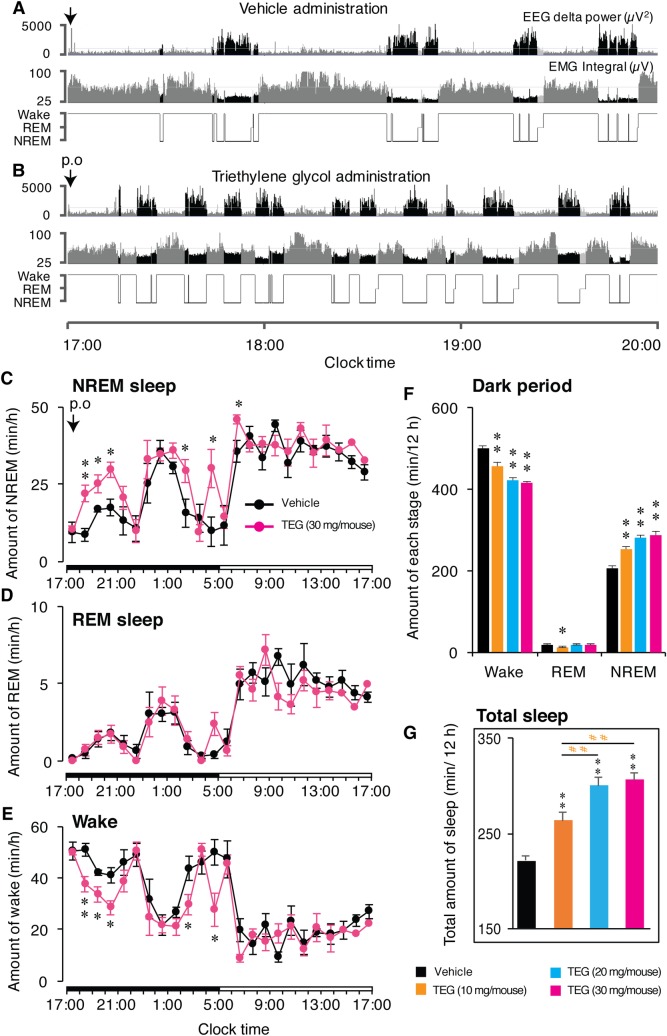
Dose-dependent increase in NREM sleep and decrease in wakefulness by *p*.*o*. administration of TEG in mice. (A, B) Typical examples of EEG delta power (0.5–4 Hz), EMG integral, and hypnograms of a mouse after *p*.*o*. administration of vehicle (A) or TEG (B). Hypnograms represent concatenated 10-sec epochs of EEG/EMG activity, scored as wake, REM, and NREM sleep. Three hours after *p*.*o*. administration are shown. Wake, REM are shown in gray while NREM sleep shown in black. (C, D and E) Hourly plots of NREM, (C), REM sleep (D) and wake (E) in mice after *p*.*o*. administration of vehicle (black circles) and TEG (30 mg/mouse; magenta circles). Significant increase in NREM sleep and decrease in wake are apparent in the graphs at least up to 4 h after TEG administration. Black and white horizontal bars indicate 12 h dark and 12 h light period. (F) Total amount of wake, REM, and NREM sleep over 12 h dark period after vehicle (black bars) and various doses of TEG (colored bars) administration. There was a dose-dependent increase in NREM sleep with concomitant decrease in wake. (G) Graph shows total amount of sleep during dark phase (12 h) following vehicle (black bar) and various doses of TEG (color bars) administration. All administrations were done at the onset of dark period (17:00 h). Data presented as mean ± SEM; n = 6; *p≤0.05, **p≤0.01 vs vehicle by using paired t-test for time course data (C, D and E), and one-way ANOVA followed by least square difference (LSD) post-hoc test for dose-response data (F and G). # #p≤0.01 vs TEG (10 mg/head).

Previously, TEG has been identified as an active component present in water extracts of Ashwagandha leaves (10). Similarly, cyclodextrin extract also had high percentage of TEG. We then investigated the sleep-inducing potential of TEG by *p*.*o*. administration in mice. Typical EEG delta power traces, EMG integral and hypnograms of a mouse after *p*.*o*. administration of vehicle (Fig 2A) or TEG (Fig 2B). Whereas the animal remained mainly awake after vehicle administration, TEG-treated animals fell asleep within few minutes after the administration. EEG traces during both vehicle and TEG treatment showed clear increase in delta power, characteristic to NREM sleep. TEG treated animal showed increased amount of delta power bouts compared to vehicle. EMG integral decreased in power corresponding to the increase in EEG delta power (Fig 2A and 2B). Time course analysis of hourly amounts of NREM, REM sleep and wake revealed that *p*.*o*. administration of TEG induced NREM sleep (Fig 2C) as compared to vehicle administration without change in REM sleep (Fig 2D), and with a concomitant decrease in wake (Fig 2E). This increase in NREM was statistically significant up to 4 h after administration. When we tested dose dependency, TEG-dependent NREM sleep was increased from 204.6±5.1 min/12 h after vehicle to 251.1±8.8, 281.1±6.9 and 287.9±5.4 min/12 h after administration of TEG at doses of 10, 20 and 30 mg/mouse, respectively, during dark phase (Fig 2F). Total amount of sleep, that was calculated by adding REM and NREM sleep (Fig 1G), and total percentage of sleep amount (S1 Fig) during dark phase, also showed dose-dependent increase. Total amount of wake was concomitantly decreased with increasing TEG dose (from 498.5±4.3 min/12 h to 413.6±7.1 min/12 h; Fig 2F). Similarly, dose-dependent decrease in total wake percentage was apparent after administration of increasing doses of TEG (S1 Fig). The amounts of REM and NREM sleep induced by TEG administration were comparable to that of positive control (doxepin; 4.5 μg/mouse; S2 Fig). Next, we adopted cage change strategy, whereby, mice were transferred to clean cages, to induce sleep deprivation for 1–2 h. We observed that TEG failed to induce changes in wake, REM, or NREM sleep under the condition of brief sleep deprivation (S3 Fig).

### TEG decreased NREM sleep onset latency and induces physiological sleep

**Fig 3:**
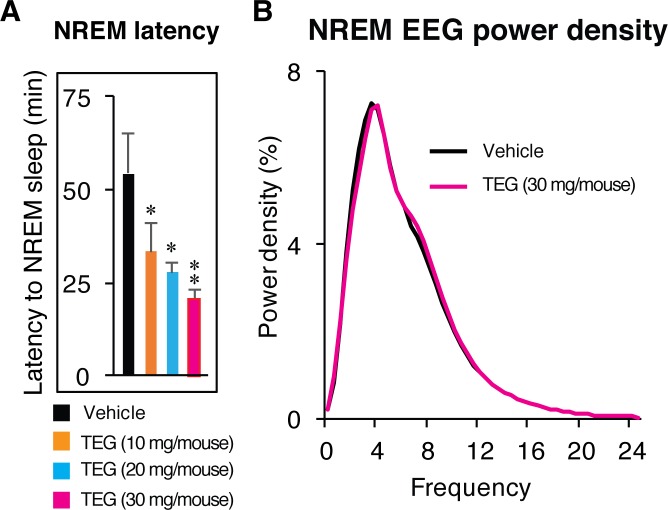
TEG decreased NREM sleep onset latency and induces physiological sleep. (A), Graph shows changes in NREM sleep onset latency after vehicle (black bar) and various doses of TEG (color bars) administration. (B), Graph shows NREM EEG power density over 12 h dark phase following vehicle (black line) and TEG (magenta line) administration in mice. Data presented as mean ± SEM; n = 6; *p≤0.05, **p≤0.01 vs vehicle by one-way ANOVA followed by least square difference (LSD) post-hoc test.

NREM sleep onset latency was calculated by observing appearance of at least two consecutive NREM epochs following vehicle or TEG administration. We observed significant decrease in NREM onset latency with increasing doses of TEG (Fig 3A). EEG power density of REM (S4 Fig) and NREM (Fig 3B) during 12 h dark phase was indistinguishable between vehicle and TEG treatment, indicating that TEG administration induced physiologically similar sleep (which can be defined as naturally occurring sleep in healthy individuals, and can be assessed by means of power density as a marker for the deepness of sleep) without affecting EEG power density. No abnormalities in EEG pattern or sleep architecture, such as sleep rebound or sleep loss, was observed during 24 h period following initial effect of TEG on NREM sleep.

### TEG induces sleep by increasing number of NREM episodes and by decreasing wake episode duration

**Fig 4:**
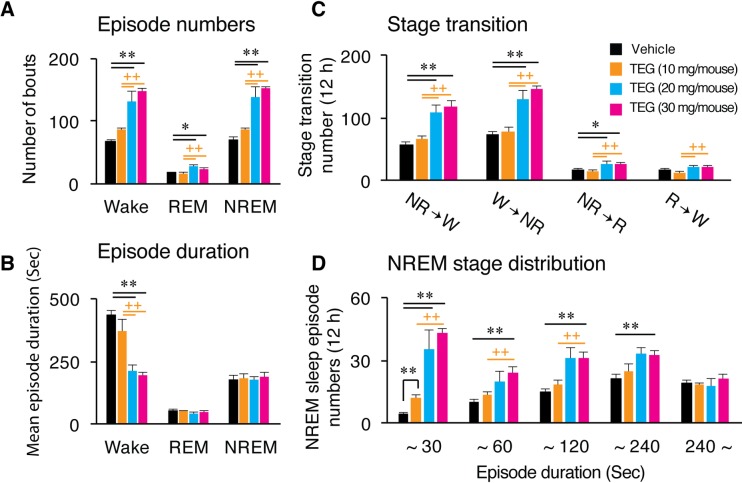
TEG induces sleep by targeting sleep generation mechanism. Graphs represent changes in number of episodes (A), stage duration (B) of wake, REM and NREM sleep during 12 h dark phase after *p*.*o*. administration of vehicle (black bars) and various doses of TEG (colored bars). (C) Stage transition between wake, REM, and NREM sleep in mice after vehicle or TEG treatment. (D) NREM sleep stage distribution was calculated by averaging various NREM episodes within specific time durations, in mice after vehicle and TEG administration. Data presented as mean ± SEM; n = 6; *p≤0.05, **p≤0.01 vs vehicle; **+**p≤0.05, **++**p≤0.01 vs 10 mg/mouse TEG, by using one-way ANOVA followed by least square difference (LSD) post-hoc test. NR: NREM sleep; W: Wake; R: REM sleep.

The sleep-wake architecture and quality was evaluated by calculating episode numbers, episode duration, stage transitions and NREM sleep stage distribution during dark phase. The episode number of NREM sleep (92.5±6.60) and wake (90.3±7.16) were significantly increased after treatment of TEG (30 mg/mouse) compared to vehicle (74.8±5.68 & 74.7±5.81, respectively; Fig 4A). TEG treatment also decreased the mean duration of wake episodes (284.7±24.67 sec/episode) compared to vehicle treatment (401.0±29.55 sec/episode; Fig 4B). Further, there was significant increase in number of transitions from wake to NREM (89.3±7.16/12 h) and from NREM to wake (70.5±7.20/12 h) after TEG treatment compared to vehicle (73.7±5.81 & 56.7±5.19/12 h, respectively; Fig 4C). TEG treatment increased the number of short NREM sleep episodes (<240 sec) leaving long episodes (>240 sec) unaffected (Fig 4D).

## Discussion

The increase in sleep following administration of Ashwagandha leaf extract was also supported by Ayurveda (Traditional medicine system of India). Whereby, ashwagandha powder (root, leaves or whole plant), induces sleep in humans on oral consumption. Further, oral administration of Ashwagandha root extract induced sleep in rats [16, 17]. Hence, we dissected out the role of various know components of Ashwagandha in sleep regulation. Withaferin-A and withanone (withanolides), are the major biologically active constituents of Ashwagandha leaves [22, 23] and believed to be involved in majority of biological functions of Ashwagandha. Withanolides are involved in various bodily functions such as anti-cancer activity [22], neuroprotection and anti-stress activity [24], recovery from amnesia [14], anti-mutagenic [25]. However, in our study, ethanol extract that contain high ratio of withaferin A and withanone, failed to induce sleep in mice, indicating that withanolides might not be involved in sleep promotion (Fig 1A). However, specified withanolides need to be tested directly, before completely ruling out withanolides and their derivatives for their sleep-inducing property. Sleep promotion was observed by water extracts that contained TEG and only a low level of withanolide (Fig 1B) and TEG itself (Fig 2). TEG administration increased sleep in a dose-dependent manner (Fig 2). Recently, anticancer activity of TEG and its involvement in neuroprotection has been reported [15, 26]. As shown in Fig 2G, TEG administration did not alter the NREM EEG power density, implying that quality of sleep was not affected by TEG administration and that TEG-induced sleep was physiological. In contrast, benzodiazepines affects the quality of sleep and reduced EEG power [27]. Hence, TEG can be a better alternative to currently available drugs, because it promotes physiological sleep (naturally occurring sleep in healthy individuals).

Sleep regulation has two components *viz* sleep generation, which is signified by number or frequency of NREM episodes, and sleep maintenance reflected in terms of NREM episode duration. TEG increased frequency of NREM sleep episodes suggesting that TEG has potential to generate sleep and that TEG increases NREM sleep by frequently entering into sleep via targeting sleep generation mechanism. Moreover, TEG promoted sleep by increasing the number of shorter episodes of NREM sleep (Fig 3D), suggesting that TEG is able to generate sleep more frequently, thus increasing total amount of NREM sleep.

TEG is primarily used for industrial purpose and very little is known about its usefulness to the biological systems. Available information is limited to animal studies, except few case reports of accidental consumption of TEG. Various animal studies showed little or no TEG toxicity, suggesting that its lower volumes are safe in biological system, but higher volumes could be dangerous [28]. A reported case of accidental TEG consumption resulted in coma and metabolic acidosis, however, treated via ethanol infusion [29]. Another case of TEG poisoning was treated by using 4-methylpyrazole [30]. These incidences, however, clearly suggests that toxicological properties of TEG need to be studied in detail before its use is advised in humans. Study on TEG metabolism suggests that maximum portion of TEG (91–98%) following administration in rats and rabbits, eliminated in urine (84–94%), and minor portion via feces and expiration. On metabolic oxidation it produces amonocarboxylic acid, ethylenedioxydiacetic acid and oxalic acid [31]. Almost nothing is known about TEG’s absorption or blood levels, whether or not it crosses blood brain barrier and its target organ.

## Conclusion

Insomnia and poor quality of sleep results in chronic sleep loss that is associated with various other sleep and metabolic disorders. Unlike conventional therapy available to treat insomnia, those develop dependency and side effects, we were interested to identify a natural compound with sleep-inducing potential. In this study, we demonstrated that TEG, which is also an active component of Ashwagandha leaves, is a potent sleep-inducing small molecule. Ashwagandha leaf or root crude powder itself is able to enhance the quality of sleep. Here we showed that a sleep-promoting active component present in Ashwagandha leaves is TEG and validated the sleep inducing potential of TEG by animal experiments. Further studies are needed to delineate its molecular targets and sleep-promoting pathways.

## Supporting information

S1 Fig TEG decreased total wake percentage and increased total sleep percentage. Graph shows changes total wake (A) and total sleep (B) percentage during 12 h dark phase after vehicle (black bar) and various doses of TEG (color bars) administration in mice. Data presented as mean ± SEM; n = 6; *p≤0.05, **p≤0.01 vs vehicle, and # #p≤0.01 vs TEG (10 mg/head), by one-way ANOVA followed by least square difference (LSD) post-hoc test.
(PDF)

S2 Fig TEG induced NREM sleep was comparable to positive control (doxepin). Graph shows time course changes in NREM sleep after TEG (magenta line) and doxepin (green line) administration in mice. Data presented as mean ± SEM; n = 6; *p≤0.05, **p≤0.01 vs vehicle by one-way ANOVA followed by least square difference (LSD) post-hoc test.
(PDF)

S3 Fig TEG failed to induce changes in NREM sleep in sleep deprived mice. Vehicle or TEG administration was immediately followed by change of mice cage, to induce wakefulness. Graph shows time course changes in REM (upper graph) and NREM (lower graph) sleep after vehicle (gray line) and TEG (blue line) administration **in mice.** Data presented as mean ± SEM; n = 6; statistical test applied was paired t-test.
(PDF)

S4 Fig TEG induced changes in REM sleep power density. Graph shows changes in REM sleep power density after vehicle (black line) and TEG (magenta line) administration in mice.
(PDF)
