# Supplementary material for: Triethylene glycol, an active component of Ashwagandha (Withania somnifera) leaves, is responsible for sleep induction
Source: PLoS One. 2017 Feb 16;12(2):e0172508. doi: 10.1371/journal.pone.0172508 (PMC5313221; doi:10.1371/journal.pone.0172508)
Supplement: S1 Fig — Graph shows changes total wake (A) and total sleep (B) percentage during 12 h dark phase after vehicle (black bar) and various doses of TEG (color bars) administration in mice. Data presented as mean ± SEM; n = 6; *p≤0.05, **p≤0.01 vs vehicle, and # #p≤0.01 vs TEG (10 mg/head), by one-way ANOVA followed by least square difference (LSD) post-hoc test. (PDF) [file pone.0172508.s001.pdf]

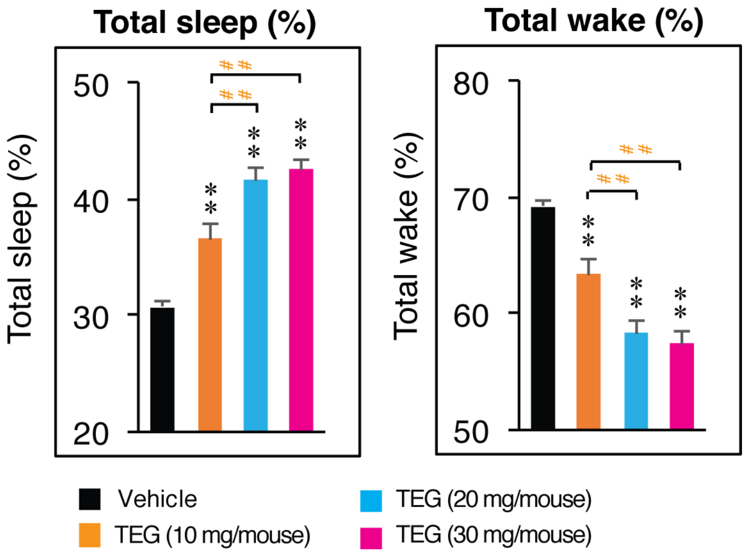

S1 Fig. TEG decreased total wake percentage and increased total sleep percentage.

Graph shows changes total wake (A) and total sleep (B) percentage during 12 h dark phase after vehicle (black bar) and various doses of TEG (color bars) administration in mice. Data presented as mean ± SEM; n=6; *p≤0.05, **p≤0.01 vs vehicle , and # #p≤0.01 vs TEG (10 mg/head), by one-way ANOVA followed by least square difference (LSD) post-hoc test.
