# Supplementary material for: Triethylene glycol, an active component of Ashwagandha (Withania somnifera) leaves, is responsible for sleep induction
Source: PLoS One. 2017 Feb 16;12(2):e0172508. doi: 10.1371/journal.pone.0172508 (PMC5313221; doi:10.1371/journal.pone.0172508)
Supplement: S2 Fig — Graph shows time course changes in NREM sleep after TEG (magenta line) and doxepin (green line) administration in mice. Data presented as mean ± SEM; n = 6; *p≤0.05, **p≤0.01 vs vehicle by one-way ANOVA followed by least square difference (LSD) post-hoc test. (PDF) [file pone.0172508.s002.pdf]

Comparision of sleep-wake after doxipin (positive control) and TEG administration

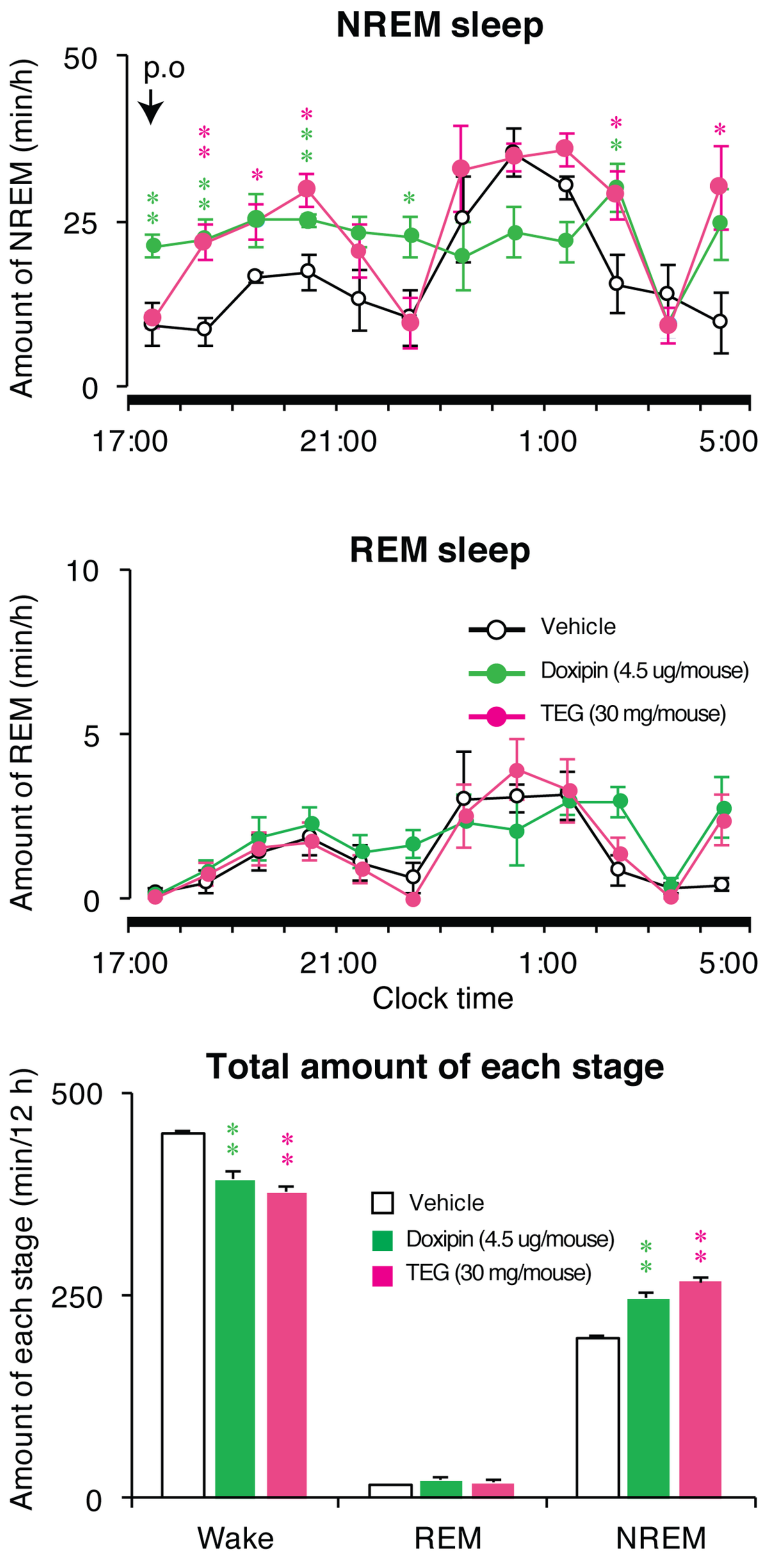

S2 Fig. TEG induced NREM sleep was comparable to positive control (doxepin). Graph shows time course changes in NREM sleep after TEG (magenta line) and doxepin (green line) administration in mice. Data presented as mean ± SEM; n=6; *$p \leq 0.05$, **$p \leq 0.01$ vs vehicle by one-way ANOVA followed by least square difference (LSD) post-hoc test.
