# Supplementary material for: Triethylene glycol, an active component of Ashwagandha (Withania somnifera) leaves, is responsible for sleep induction
Source: PLoS One. 2017 Feb 16;12(2):e0172508. doi: 10.1371/journal.pone.0172508 (PMC5313221; doi:10.1371/journal.pone.0172508)
Supplement: S3 Fig — Vehicle or TEG administration was immediately followed by change of mice cage, to induce wakefulness. Graph shows time course changes in REM (upper graph) and NREM (lower graph) sleep after vehicle (gray line) and TEG (blue line) administration in mice. Data presented as mean ± SEM; n = 6; statistical test applied was paired t-test. (PDF) [file pone.0172508.s003.pdf]

Sleep-wake stages after TEG administration during mild sleep deprivation (cage change)

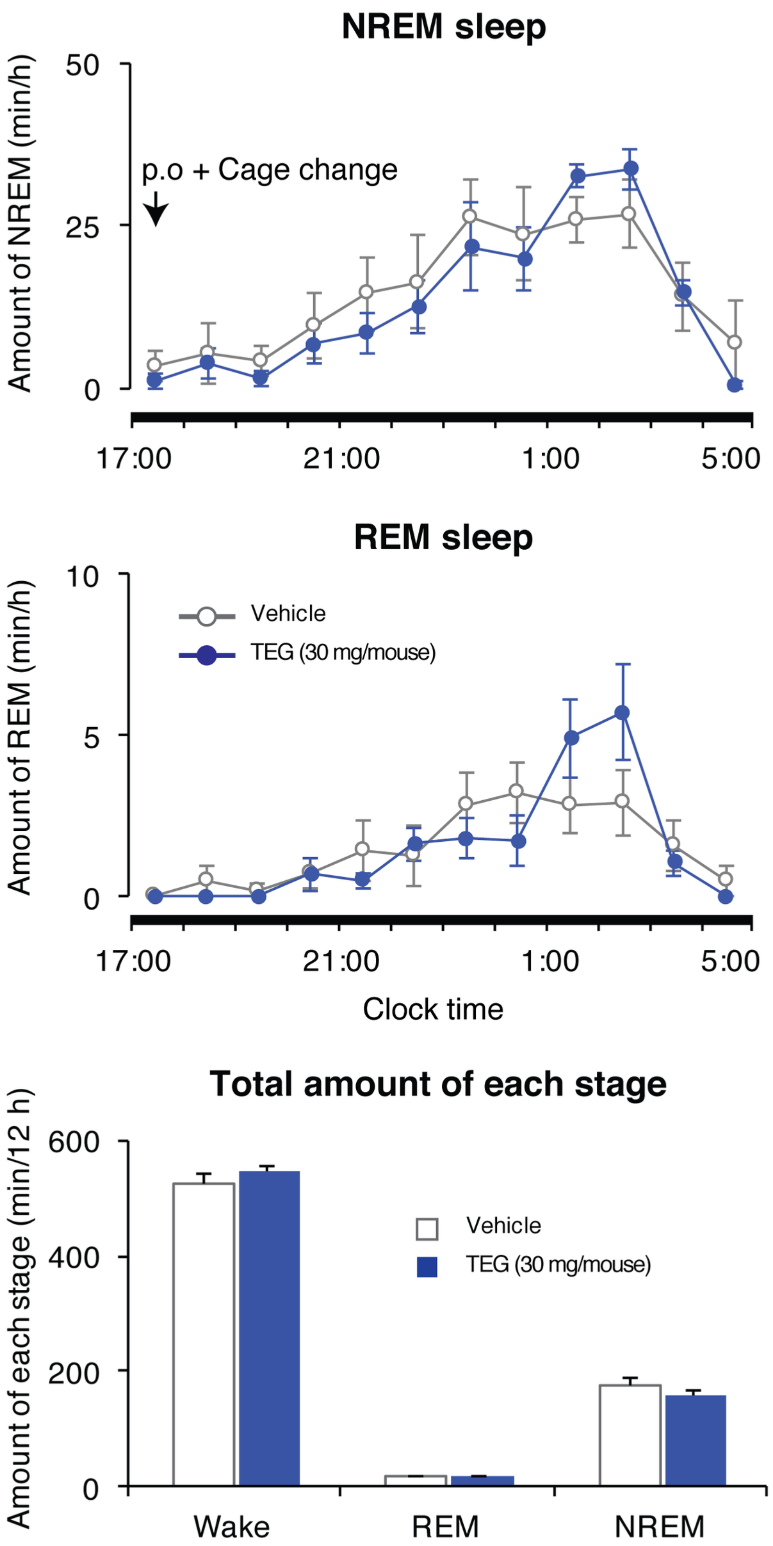

S3 Fig. TEG failed to induce changes in NREM sleep in sleep deprived mice. Vehicle or TEG administration was immediately followed by change of mice cage, to induce wakefulness. Graph shows time course changes in REM (upper graph) and NREM (lower graph) sleep after vehicle (gray line) and TEG (blue line) administration **in mice.** Data presented as mean ± SEM; n=6; statistical test applied was paired t-test.
