# Supplementary material for: Triethylene glycol, an active component of Ashwagandha (Withania somnifera) leaves, is responsible for sleep induction
Source: PLoS One. 2017 Feb 16;12(2):e0172508. doi: 10.1371/journal.pone.0172508 (PMC5313221; doi:10.1371/journal.pone.0172508)
Supplement: S4 Fig — Graph shows changes in REM sleep power density after vehicle (black line) and TEG (magenta line) administration in mice. (PDF) [file pone.0172508.s004.pdf]

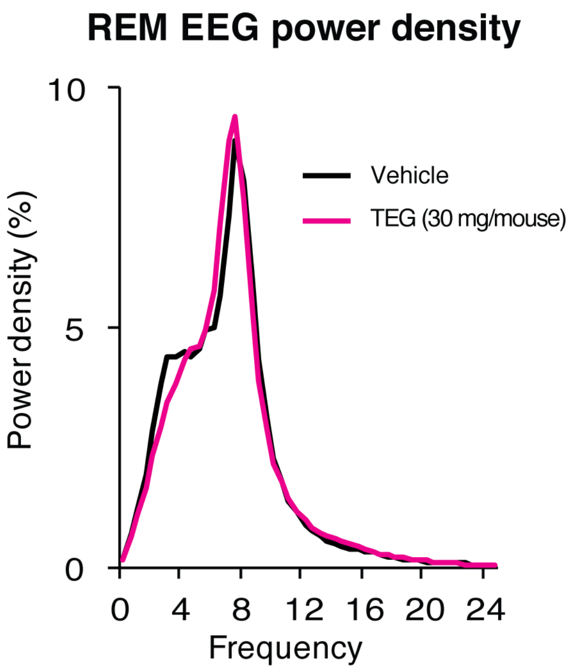

S4 Fig. TEG induced changes in REM sleep power density. Graph shows changes in REM sleep power density after vehicle (black line) and TEG (magenta line) administration in mice.
